# NF-κB-mediated lncRNA AC007271.3 promotes carcinogenesis of oral squamous cell carcinoma by regulating miR-125b-2-3p/Slug

**DOI:** 10.1038/s41419-020-03257-4

**Published:** 2020-12-12

**Authors:** Ze-nan Zheng, Guang-zhao Huang, Qing-qing Wu, Heng-yu Ye, Wei-sen Zeng, Xiao-zhi Lv

**Affiliations:** 1 Department of Oral & Maxillofacial Surgery, NanFang Hospital, Southern Medical University, Guangzhou, People’s Republic of China; 2 Department of Cell Biology, School of Basic Medical Science, Southern Medical University, Guangzhou, People’s Republic of China

**Keywords:** Metastasis, Oral cancer

## Abstract

Oral squamous cell carcinoma (OSCC) is the most common oral cancer. The molecular mechanisms of this disease are not fully understood. Our previous studies confirmed that dysregulated function of long non-coding RNA (lncRNA) AC007271.3 was associated with a poor prognosis and overexpression of AC007271.3 promoted cell proliferation, migration, invasion, and inhibited cell apoptosis in vitro, and promoted tumor growth in vivo. However, the underlying mechanisms of AC007271.3 dysregulation remained obscure. In this study, our investigation showed that AC007271.3 functioned as competing endogenous RNA by binding to miR-125b-2-3p and by destabilizing primary miR-125b-2, resulted in the upregulating expression of Slug, which is a direct target of miR-125b-2-3p. Slug also inhibited the expression of E-cadherin but N-cadherin, vimentin, and β-catenin had no obvious change. The expression of AC007271.3 was promoted by the canonical nuclear factor-κB (NF-κB) pathway. Taken together, these results suggested that the classical NF-κB pathway-activated AC007271.3 regulates EMT by miR-125b-2-3p/Slug/E-cadherin axis to promote the development of OSCC, implicating it as a novel potential target for therapeutic intervention in this disease.

## Introduction

Oral squamous cell carcinoma (OSCC) is the most common type of oral cancer^1^. Tobacco, alcohol, betel quid, and human papillomavirus infection are the major risk factors for OSCC^2^. Despite recent remarkable advances in the treatment of OSCC in surgery, chemotherapy, radiotherapy and targeting therapy, the 5-years survival rate is still <60% owing to tumor recurrence and metastasis^3,4^. Therefore, understanding the detailed mechanisms of OSCC tumorigenesis and development will facilitate the establishment of new effective therapeutic alternatives in order to improve the curative effect and life quality.

LncRNAs are a group of genes longer than 200 nucleotides, which were considered as “functionless genes” in the past^5^. Over the past decade, numerous studies have shown that lncRNAs actually play important roles in various diseases, including cancers^6,7^. LncRNAs can function as tumor suppressors or oncogenes, transcriptional regulation, histone modification elements, splicing, and so on, which would promote or inhibit the OSCC progression^8–10^.

AC007271.3 is a 382-nucleotides-long lncRNA located on chromosome 2. Our previous study identified that AC007271.3 was significantly upregulated in OSCC than in adjacent normal tissues (ANTs) by microarray. Higher expression of AC007271.3 in serum was related with clinical stage and poor prognosis^11^. Further study showed that AC007271.3 mainly enriched in the cytoplasm and promoted OSCC cells proliferation, migration, invasion, and inhibited cell apoptosis in vitro, and promoted tumor growth in vivo^12^. However, the underlying mechanisms of AC007271.3, promoting OSCC carcinogenesis remain to be excavated.

MicroRNAs (miRNAs) are a group of non-coding RNAs with a length of ~22 nucleotides, which can direct the RNA-induced silencing complex to suppress the mRNA translation or degrade them by targeting the 3′-untranslated region (3′-UTR) of mRNA^13^. Accumulating evidence proved that miRNAs were related to cancers, and several miRNAs have been researched as therapeutic molecular^14^. Recent studies indicated that lncRNAs located in the cytoplasm may play as competing endogenous RNA (ceRNA) of miRNAs^15^. This phenomenon was widely discovered^16,17^ and also reported in OSCC^18,19^.

According to the location of AC007271.3, we speculated that it could play as a ceRNA of miRNAs. Further investigation verified that AC007271.3 increased Slug expression by regulating miR-125b-2-3p and promoted the migration and invasion in OSCC cells. What’s more, classical nuclear factor-κB (NF-κB) pathway was found to activate the expression of AC007271.3. Our findings provided a novel insight into the mechanisms of AC007271.3 in OSCC development, which suggested AC007271.3 could be a possible target for OSCC therapy.

## Materials and methods

### Ethics statement and tissue samples

The OSCC tumor tissues and matched ANTs in this study were collected from 82 patients with OSCC under surgery at the Department of Oral and Maxillofacial Surgery, Nanfang Hospital, Guangzhou, Guangdong Province after obtaining informed consent. The diagnosis was pathologically confirmed by the Department of Pathology, Nanfang Hospital. ANTs located at least 1.5 cm from the edge of the tumor were defined as normal control. This study was approved by Research Scientific Ethics Committee of NanFang Hospital (NFEC-2018-027).

### Cell culture

The human tongue squamous cell lines (SCC9 and SCC15), normal human oral keratinocyte cell line (HOK), and human embryonic kidney cell line (HEK 293 T) were obtained from the Institute of Antibody Engineering, Southern Medical University (Guangzhou, China). All cells were cultured in Dulbecco’s Modified Eagle’s Medium (DMEM) (Gibco, NY, USA) with 10% Fetal bovine serum (ExCell Bio, Shanghai, China) and 100U/ml Penicillin-Streptomycin (Invitrogen, CA, USA) at 37 °C in a humidified 5% CO_2_.

### Expression plasmid, RNA oligonucleotides, and transfection

The AC007271.3 overexpression vector pcDNA3.1(+)-AC007271.3 and its corresponding control vector pcDNA3.1(+), specific siRNAs targeting AC007271.3 (si-AC007271.3), Slug (si-Slug 1, si-Slug 2, si-Slug 3) and the scramble negative control siRNA (si-AC007271.3 NC, si-Slug NC) were obtained from Sangon Biotech Corp., Ltd. (Shanghai, China). The miR-125b-2-3p inhibitor, miR-125b-2-3p mimic, and the negative control (miR-125b-2-3p mimic NC, miR-125b-2-3p inhibitor NC) were purchased from RiboBio Corp, Ltd. (Guangzhou, China). Transfections were performed with Lipofectamine 3000 reagent (Invitrogen, CA, USA) following the manufacturer’s protocols.

### RNA extraction and qRT-PCR

Total cellular RNA from the cells was extracted by RNA isolater Total RNA Extraction Reagent (Vazyme, Nanjing, China) following the manufacturer’s instructions. HiScript QRT SuperMix for qPCR (+gDNA wiper) (Vazyme, Nanjing, China) and miRNA 1st Strand cDNA Synthesis Kit (by stem-loop) (Vazyme, Nanjing, China) were used to generate cDNA. With ChamQ® SYBR® qPCR Master Mix (Vazyme, Nanjing, China), quantitative real-time polymerase chain reaction (qRT-PCR) was performed on the Real-Time PCR detection system (Applied Biosystems, CA, USA). All expression levels were normalized against the GAPDH or U6 (for miRNA) mRNA level. The primer sequences are listed in Supplementary Table S1.

### mRNA stability assay

The pcDNA3.1(+)-AC007271.3 plasmids were transfected into OSCC cells as described above. After 48 hours, actinomycin D (Sigma-Aldrich, St. Louis, MO, USA) was added to the culture medium at a concentration of 5 μg/mL, followed by incubation for 0 hour, 2 hours, 4 hours, 6 hours, or 8 hours. Pri-miR-125b-2 mRNA stability in the actinomycin D treatment group was analyzed by qRT-PCR.

### Dual-luciferase reporter assay

PmiRGLO dual-luciferase miRNA Target Expression Vector (Promega, WI, USA) was used to construct dual-luciferase reporter and quantitatively evaluate the translational regulation activity of miR-125b-2-3p. The features list and map of this empty vector were showed in Supplementary Fig. S1. The predicted binding sites between miR-125b-2-3p and AC007271.3 (or the 3′-UTR of Slug mRNA) were inserted into the multiple cloning site region to construct the wild-type vectors (AC007271.3-WT, Slug-WT). Then the mutant type vectors (AC007271.3-MUT, Slug-MUT) were obtained from the wild-type vectors by replacing the targeted sites (Replaced ACTTGTG with TCATCTC for AC007271.3-MUT, replaced ACTTGTGA with ACTGTCGA for Slug-MUT). All vectors were constructed by TsingKe Biological Technology Corp, Ltd. (Beijing, China). After co-transfecting with miR-125b-2-3p mimic into HEK 293 T cells for 48 hours, the Luc-Pair Dual-Luciferase Assay Kit 2.0 (GeneCopoeia, MD, USA) was used to detect the firefly and renilla luciferase activity. Renilla luciferase activity was used as control. For the core promoter identification, pGL3 vectors which contain the promoter fragments and phRL-TK vector were co-transfected into HEK 293 T cells for 48 hours and the following steps were the same.

### RNA pull‐down assay

Purified RNAs were biotin-labeled using Pierce RNA 3′-End Desthiobiotinylation Kit (Thermo Fisher Scientific, CA, USA). Biotin-labeled wild-type miR-125b-2-3p (miR-125b-2-3p-Bio), Biotin-labeled mutant type miR-125b-2-3p (miR-125b-2-3p-Bio-MUT) and negative control (NC-Bio) were incubated with SCC9 cell lysates overnight at 4 °C. Magnetic beads were added to each mixture for 1 hour at room temperature. The eluted RNAs were detected by qRT-PCR.

### Western blot and antibody

Protein obtained from cells or tissues were separated on 10% sodium dodecyl sulphate–polyacrylamide gel (16 mA, 1.5 h) and then transferred to polyvinylidene fluoride membrane (100 V, 1.5 h) (Bio-Rad Laboratories). After blocking (5% non-fat dry milk in phosphate-buffered saline (PBS) Tween) for 2.5 hours at room temperature, the primary antibodies (Supplementary Table S2) were used for incubation at 4 °C overnight and the secondary antibodies were incubated one hour at room temperature. ECL reagent (Millipore, Bedford, MA) was used for chemiluminescence. Molecular weight markers (Thermo Fisher Scientific, CA, USA, #26616) was used as size standards in this assay.

### Cell migration and matrigel invasion assay

OSCC cells transfected with RNA oligonucleotides or plasmid for 48 h were digested and resuspended in DMEM culture. In all, 5 × 10^4^ cells were seeded into the upper chambers of 8-μm transwell inserts (Corning, NY, USA), and 500 μl DMEM containing 20% FBS was added to the lower chamber. After 36 hours’ incubation, the membranes were fixed with methanol and stained with 0.1% crystal violet. Removed the cells on the top of the membranes and then counted the number of cells attached to the membranes under an inverted microscope. For invasion assay, matrigel (Corning, NY, USA) was diluted with DMEM (1:10) and covered the top membranes before cells seeding. Other steps were the same as migration assay.

### H&E and IHC staining

For histological examination, tissues were fixed with 4% paraformaldehyde at room temperature for 24 hours and washed with 70% alcohol. All tissues were embedded in paraffin and cut into sections (4 µm thick). Hematoxylin and eosin were used for Hematoxylin and Eosin (H&E) staining. For immunohistochemical (IHC) staining, after dewaxing, washing, rehydration, antigen retrieval, and endogenous peroxidase blocking following the manufacturer’s protocols. Antibody was used to incubate at 4 °C overnight. Secondary biotinylated conjugated goat anti-rabbit antibody was incubated at room temperature for 30 mins. DAB (3,3’-diaminobenzidine) were used as chromogens and hematoxylin used as counterstains. Images were acquired under the light microscope (Leica, DM 2500, Wetzlar, Germany). The comparison of staining results between tumor tissues and ATNs was performed by two independent pathologists who were blinded to the clinical information of patients. The scoring criteria of staining were as follow, first was staining intensity: 0-none staining, 1-light yellow, 2-deep yellow, or 3-brown; and the second criterion was staining cells proportion: 0 (<5%), 1 (5–25%), 2 (25–50%), 3 (51–75%), or 4 (>75%). The product of the two scores was considered as the final score. The final scores were divided into two levels: 0–5 (Slug low expression) and >5 (Slug high expression).

### Animal experiment

Lentivirus-NC and lentivirus-AC007271.3 were purchased from Obio Technology Corp., Ltd. (Shanghai, China) and transfected into SCC9 according to the operating instructions. Puromycin was used to select the AC007271.3 stably expressed cells (SCC9-AC007271.3) and its control cells (SCC9-NC). Five-weeks-old female BALB/c nude mice were randomly divided into three groups (Blank, SCC9-NC and SCC9-AC007271.3). Approximately 1 × 10^6^ SCC9-AC007271.3 cells or SCC9-NC cells were resuspended in 500 μL of PBS and injected via tail vein. Injection of 500 μL of PBS without cells suspension was regarded as blank group. After 8 weeks, all mice were killed, and the visible lung metastases were counted. Lung metastases were excised for HE or IHC staining. The expression of AC007271.3 and miR-125b-2-3p were detected by qRT-PCR and the expression of Slug and E-cadherin were inspected by western blot. This animal experiment was approved by Southern Medical University Experimental Ethics Committee (L2018199), and all BALB/c nude mice were purchased from the Animal Care Unit of Southern Medical University.

### AC007271.3-promoter region cloning

The sequences of the 2000bp before the 5′-UTR of AC007271.3 were regarded as promoter region and obtained from Ensemble database (http://asia.ensembl.org/index.html). According to the sequences, we designed several primers with specific restriction enzyme protection sites of KpnI and Bgl II, respectively, for amplification the fragments (−1998/−2, −1508/−2, −1000/−2, −519/−2). Genomic DNA was used as a template to amplify by KOD-Plus-Neo (TOYOBO, Osaka, Japan) amplification enzyme. The reaction conditions were set following the instruction. 1% agarose gel electrophoresis was performed to select the bands in correct size and confirmed through sequencing (Sangon, Shanghai, China). The pGL3-Basic vector and correct fragments were digested with KpnI (TakaraBio, Dalian, China) and Bgl II (TakaraBio, Dalian, China) restriction enzymes. DNA ligation kit ver.2.1 (TakaraBio, Dalian, China) was used to connect the fragments and pGL3-Basic vector. DH5α competent cells were used for transformation. After the monoclonal colony sequence confirmation, the plasmids were extracted by using HiPure Plasmid EF Mini Kit (Magen, Guangzhou, China) for identification of core promoter of AC007271.3 by Dual-luciferase reporter assay.

### Chromatin immunoprecipitation (ChIP) assay

ChIP assay were carried out by Magna ChIP^TM^ A / G Kits (Merck, Darmstadt, German) according to the manufacturer’s protocols. In brief, SCC9 cells were crosslinked with 1% formaldehyde, glycine quenching, and lysed. The nuclear DNA was fragmented by sonication. NFKB1 antibody was used for immunoprecipitation of crosslinked protein/DNA. Protein/DNA complexes were eluted and reverse crosslinked into free DNA. Purified DNA was used for qPCR analysis to detect the enrich fragments.

### Bioinformatics analysis

Coding Potential Caculator (http://cpc.cbi.pku.edu.cn/) were performed to predict the protein-coding ability of AC007271.3. LncBase Predicted v.2 database (http://carolina.imis.athena-innovation.gr/diana_tools/web/index.php?r = lncbasev2%2Findex-predicted) were used to predict the potential miRNAs regulated by AC007271.3. TargetScan (http://www.targetscan.org/) were carried out to predict the target genes of miRNAs. The transcriptome profiling datum of OSCC were downloaded from The Cancer Genome Atlas (TCGA) database (https://portal.gdc.cancer.gov/) through GDC Data Transfer Tool. The Slug mRNA sequence data and relevant clinical information of 319 cases of OSCC and 32 cases of cancer ANTs were extracted from transcriptome profiling by R software (Version 3.6.1). The miR-125b-2-3p expression analysis results of OSCC and the GEO accession number (GSE45238) were obtained from dbDEMC 2.0 (https://www.picb.ac.cn/dbDEMC/). The miRNA sequence data of GSE45238 were downloaded from Gene Expression Omnibus (GEO) database (https://www.ncbi.nlm.nih.gov/geo/query/acc.cgi?acc=GSE45238) and extracted the expression data of miR-125b-2-3p. Gene-regulation online website (http://gene-regulation.com/pub/programs.html) and JASPAR database (http://jaspar.genereg.net/) were used for transcription factors prediction.

### Statistical analysis

All experimental assays were performed in triplicate. All data were presented as mean ± standard deviation (SD) of triplicate replicates and analyzed by SPSS statistics 20.0 (Chicago, USA). All statistical charts were manufactured in Graphpad Prism 7.0 (CA, USA). Student’s *t* test was performed to compare the differences between the two groups. The correlation between miR-125b-2-3p (or Slug) and clinicopathological features was analyzed by using Fisher’s exact tests. Pearson’s correlation coefficient analysis was used to analyze the correlation between AC007271.3 and miR-125b-2-3p. Kaplan–Meier curve was applied for detecting the comparison of overall survival in two groups.

## Results

### 1. Negative relation between AC007271.3 and miR-125b-2-3p expression

**Fig. 1:**
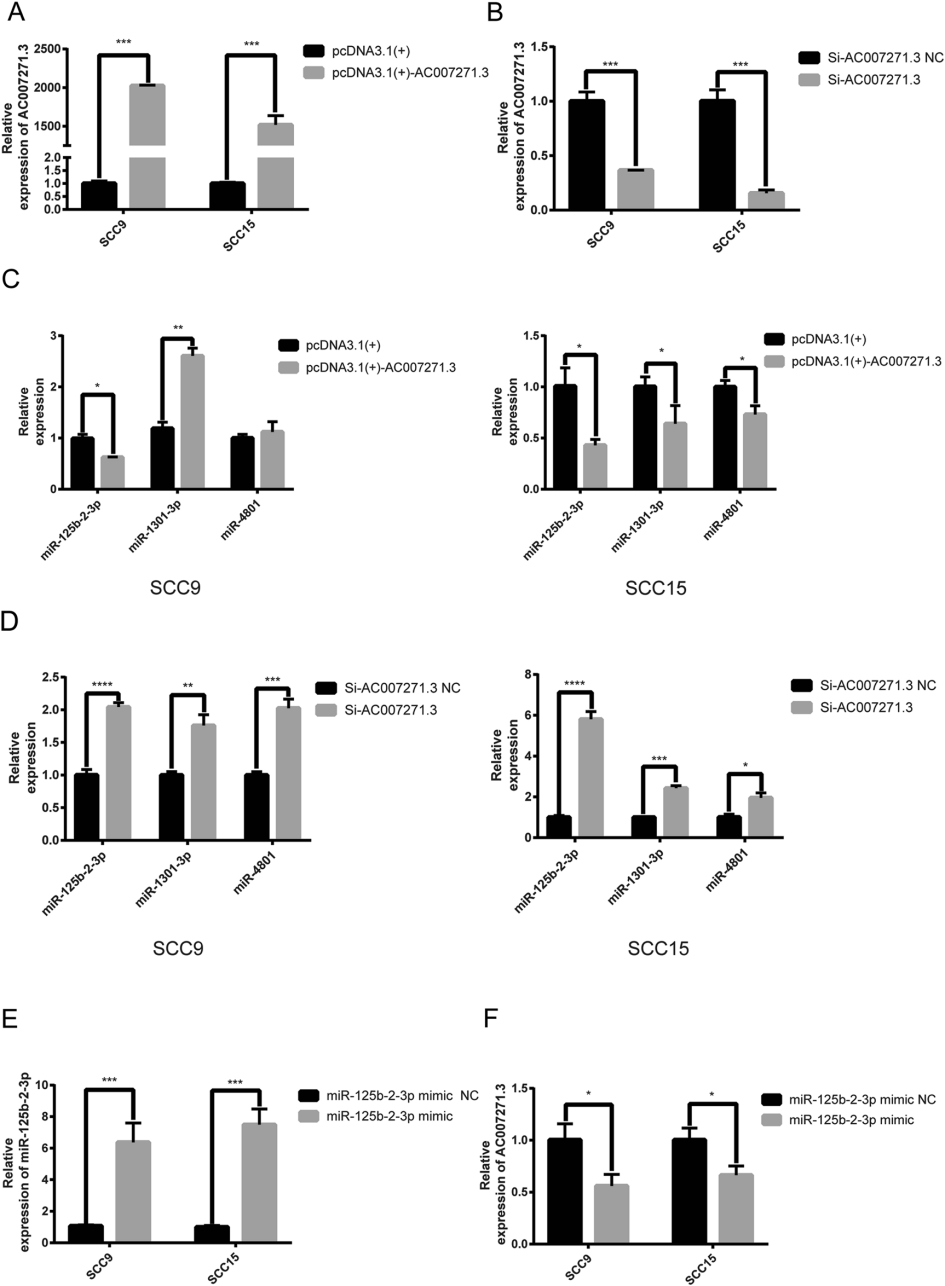
AC007271.3 and miR-125b-2-3p regulated with each other. **a** The overexpression efficiency of pcDNA3.1(+)-AC007271.3 in SCC9 and SCC15 cells. **b** The knockdown efficiency of siRNA-AC007271.3 in SCC9 and SCC15 cells. **c** The expression of three predicted miRNAs after overexpressing AC007271.3. MiR-125b-2-3p were both significantly downregulated in SCC9 and SCC15. **d** The expression of three predicted miRNAs after knocking down AC007271.3 expression. MiR-125b-2-3p were the most significantly upregulated miRNA both in SCC9 and SCC15. **e** The overexpression efficiency of miR-125b-2-3p mimic in SCC9 and SCC15 cells. **f** The upregulation of miR-125b-2-3p inhibited the expression of AC007271.3. *p < 0.05, **p < 0.01, ***p < 0.001, ****p < 0.0001.

Our previous study showed that AC007271.3 could promote the proliferation, migration, and invasion in OSCC^12^. We first predict the protein-coding potential of AC007271.3 by using Coding Potential Calculator. As exhibited in Supplementary Fig. S2, AC007271.3 had no apparent protein-coding ability. In recent years, cumulative evidence revealed that lncRNAs are vital in physiopathological processes partially by interacting with miRNAs. According to online platform LncBase Predicted v.2 database, three miRNAs (miR-4801, miR-1301-3p, miR-125b-2-3p), which contained the complementary sequences with AC007271.3 were predicted. To verify which miRNA or miRNAs were the potential target of AC007271.3, we overexpressed (Fig. 1a) or knocked down (Fig. 1b) AC007271.3 in two OSCC cell lines, respectively. The expression levels of three miRNAs were detected by qRT-PCR and the results showed that AC007271.3 could negatively regulate miR-125b-2-3p expression but induced inconsistent expression changes of miR-4801 and miR-1301-3p (Fig. 1c–d). On the other hand, overexpression of miR-125b-2-3p (Fig. 1e) resulted in a decrease of AC007271.3 (Fig. 1f), which indicated that AC007271.3 and miR-125b-2-3p could influence the expression with each other.

### 2. AC007271.3 destabilized pri-miR-125b-2 and sponged with miR-125b-2-3p

**Fig. 2:**
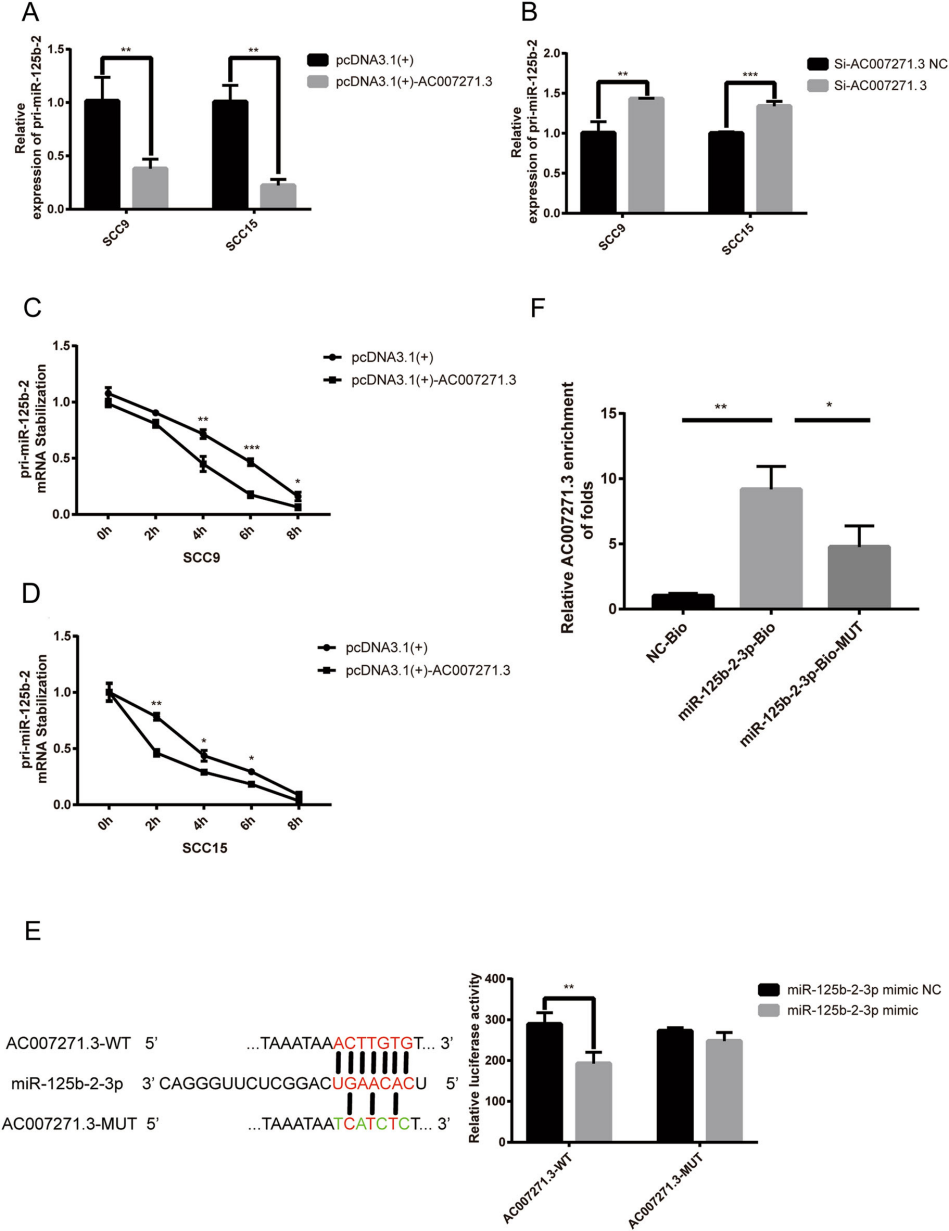
AC007271.3 interacts with mature miR-125b-2-3p and destabilizes the pri-miR-125b-2 transcript. **a** Levels of pri-miR-125b-2 after overexpressing AC007271.3 in SCC9 and SCC15. **b** Levels of pri-miR-125b-2 after knocking down AC007271.3 in SCC9 and SCC15. **c**–**d** Stability of the pri-miR-125b-2-3p mRNA after AC007271.3 overexpression were examined at different times (0 h, 2 h, 4 h, 6 h, 8 h) after treating with actinomycin D (5 μg/mL) in SCC9 and SCC15. **e** MiR-125b-2-3p overexpression reduced the relative luciferase activity in AC007271.3-WT but not in AC007271.3-MUT. **f** RNA pulldown assay indicated the direct interaction between miR-125b-2-3p and AC007271.3 in SCC9. *p < 0.05, **p < 0.01, ***p < 0.001.

Mature miR-125b-2-3p was produced by primary miRNA125b-2 (pri-miR-125b-2), which also contains the predicted complementary sequences with AC007271.3. In order to explore whether AC007271.3 could downregulate miR-125b-2-3p by decreasing pri-miR-125b-2 or not, AC007271.3 was overexpressed or knocked down in OSCC cells and the results showed that AC007271.3 was also negatively correlated with pri-miR-125b-2 (Fig. 2a, b). The mRNA stabilization assays further confirmed that overexpression of AC007271.3 enhanced the degradation of pri-miR-125b-2 (Fig. 2c, d).

Furthermore, dual-luciferase reporter assays show that a significant decrease of luciferase activity was observed in the AC007271.3 wild-type’s group but not in the mutant type’s group (Fig. 2e) and the RNA pulldown assay exhibited that higher enrichment of AC007271.3 was observed in biotinylated miR-125b-2-3p probe than in control probe or biotinylated miR-125b-2-3p mutant probe (Fig. 2f). These results further confirmed that AC007271.3 binds directly with miR-125b-2-3p.

### 3. Overexpression of miR-125b-2-3p inhibited migration and invasion in OSCC

**Fig. 3:**
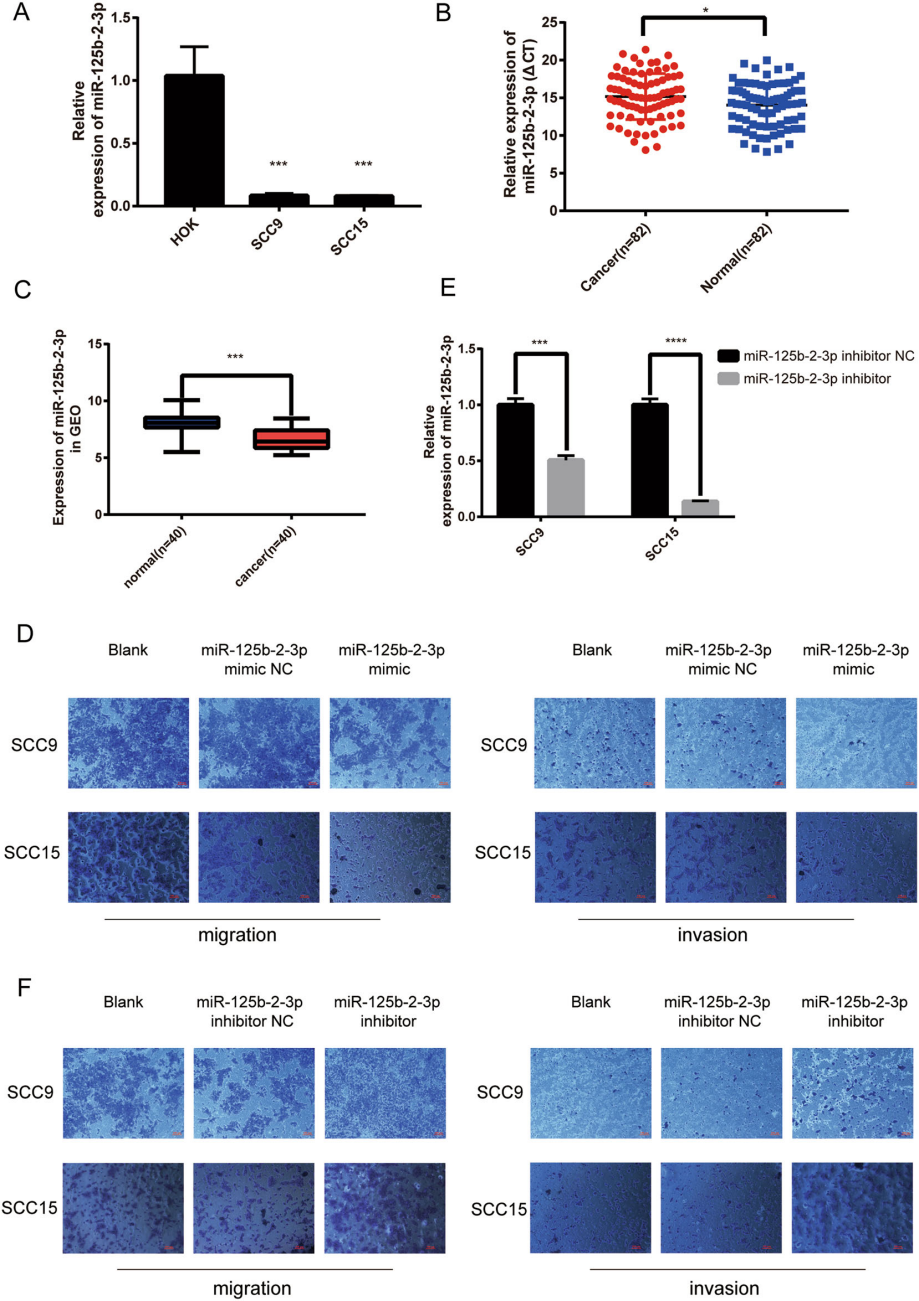
MiR-125b-2-3p regulated migration and invasion in OSCC cells. **a** Lower expression of miR-125b-2-3p was detected in two OSCC cells compared to HOK cells. **b** Differential expression of miR-125b-2-3p in 82 pairs of OSCC clinical samples (Higher Δ CT means the lower relative expression of miR-125b-2-3p). **c** The expression data of miR-125b-2-3p in 40 pairs of OSCC in GEO database. **d** Overexpressing miR-125b-2-3p inhibited the migration (left) and invasion (right) in SCC9 and SCC15. **e** The inhibition efficiency of miR-125b-2-3p inhibitor in SCC9 and SCC15 cells. **f** Inhibiting the expression of miR-125b-2-3p promoted the migration (left) and invasion (right) ability in SCC9 and SCC15. *p < 0.05, **p < 0.01, ***p < 0.001, ****p < 0.0001.

To investigate the roles of miR-125b-2-3p in OSCC carcinogenesis, first, the relative expression levels of miR-125b-2-3p in OSCC cells were detected by qRT-PCR. The results showed that the expression levels of miR-125b-2-3p were significantly lower in OSCC cells than in HOK cells (Fig. 3a). What’s more, compared with the matched ANTs, the expression levels of miR-125b-2-3p were downregulated in 82 OSCC tissues (Fig. 3b). A similar analysis result was also acquired from GEO database (Fig. 3c). We further investigated the association between miR-125b-2-3p expression and clinicopathological features of 82 OSCC patients. The results indicated that the expression of miR-125b-2-3p was negatively correlated with TNM classification (*p* = 0.0429) and lymph node metastasis (*p* = 0.0259) in OSCC patients (Supplementary Table S3).

Considered that miR-125b-2-3p was downregulated in OSCC tissues and cells, we next investigated the effects of miR-125b-2-3p overexpression on OSCC cell phenotypes. The results showed that miR-125b-2-3p mimic inhibited the ability of migration and invasion in SCC9 and SCC15 (Fig. 3D), whereas miR-125b-2-3p inhibitor (Fig. 3e) displayed the opposite results (Fig. 3f).

### 4. Slug acted as a target gene of miR-125b-2-3p

**Fig. 4:**
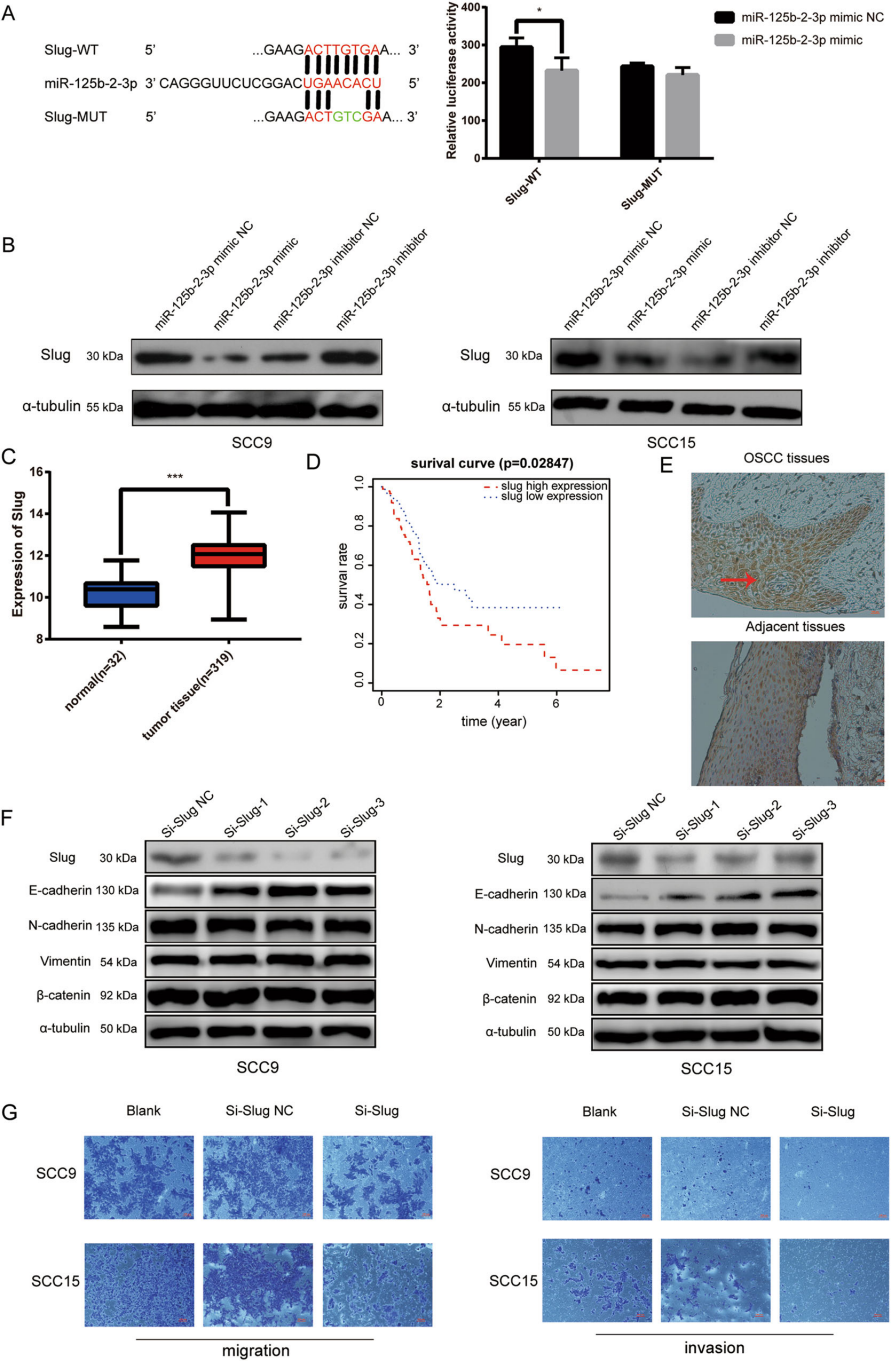
Slug was a target gene of miR-125b-2-3p and positively correlated with the malignancy of OSCC. **a** MiR-125b-2-3p overexpression reduced the relative luciferase activity in Slug-WT. **b** Overexpressing or inhibiting miR-125b-2-3p changed the expression of Slug protein in SCC9 (left) and SCC15(right). **c** The expression of Slug in OSCC (*n* = 319) and normal (*n* = 32) tissues in TCGA database. **d** Kaplan–Meier analysis were employed to estimate the relation between Slug expression and overall survival of OSCC patients. **e** Representative images of Slug expression in the OSCC tumor and adjacent non-malignant tissue analyzed by immunohistochemical. **f** After transfecting si-Slug in SCC9 and SCC15, the expression of Slug, E-cadherin, N-cadherin, vimentin, and β-catenin was detected by western blot. **g** Si-Slug inhibited the migration(left) and invasion(right) in SCC9 and SCC15. *p < 0.05, **p < 0.01, ***p < 0.001.

Plenty of evidence showed that miRNAs could target the 3′-UTR of genes’ mRNAs to suppress their translation or degrade them. To investigate the molecular mechanism of miR-125b-2-3p, we predicted the target gene of miR-125b-2-3p based on the online TargetScan V7.2 software. Slug was selected as a candidate gene for its high binding score and 8mers seed sequences (Supplementary Fig. S3). Dual-luciferase reporter assay was performed and the results showed that obviously reduced luciferase activity was observed in Slug-wild-type, whereas no remarkable change of luciferase activity in Slug-mutation type (Fig. 4a), which meant miR-125b-2-3p could bind on the 3′-UTR of Slug mRNA. Furthermore, overexpression of miR-125b-2-3p decreased the protein level of Slug, while the result was just the opposite after knocking down miR-125b-2-3p (Fig. 4b). However, Slug mRNA had no remarkable change no matter whether miR-125b-2-3p was overexpressed or low-expressed (Supplementary Fig. S4), which indicated that miR-125b-2-3p inhibit the translation of Slug mRNA.

### 5. Slug was overexpressed in OSCC and silencing Slug inhibited migration and invasion in OSCC cells

Slug is an epithelial-mesenchymal transition (EMT)-related transcription factor that could be involved in the invasion and distant metastasis of various tumor cells including OSCC^20,21^. To verified the potential biological functions of Slug in OSCC, Slug expression profiles, and corresponding clinical data of 319 OSCC patients and 32 normal controls were downloaded from TCGA database. Bioinformatics analysis revealed that Slug expression was remarkably upregulated (*p* < 0.001) (Fig. 4c) and higher expression of Slug meant poor survival and prognosis (*p* = 0.02847) (Fig. 4d). To further validate these results, the Slug expression was detected in OSCC tumor tissues and matched ANTs with immunohistochemistry. Compared with the ANTs, Slug expression was significantly higher in OSCC tumor tissues (Fig. 4e). Clinicopathological characteristics analysis showed that high expression of Slug was positively correlated with TNM classification (*p* = 0.0301), lymph node metastasis (*p* = 0.0306), and differentiation in OSCC patients (*p* = 0.0465) (Supplementary Table S4). Furthermore, we investigated the expression of Slug, β-catenin, and some EMT-related markers (E-cadherin, N-cadherin, and vimentin) after knocking down Slug by specific siRNAs (si-Slug 1, si-Slug 2, si-Slug 3) against Slug transcript in OSCC cells. E-cadherin was significantly upregulated while N-cadherin, vimentin, β-catenin had no obvious change (Fig. 4f). Finally, transwell assays indicated that the deletion of Slug could remarkably inhibit the ability of migration and invasion in OSCC cells (Fig. 4g).

### 6. AC007271.3 promoted migration and invasion via miR-125b-2-3p/Slug axis in OSCC cells

**Fig. 5:**
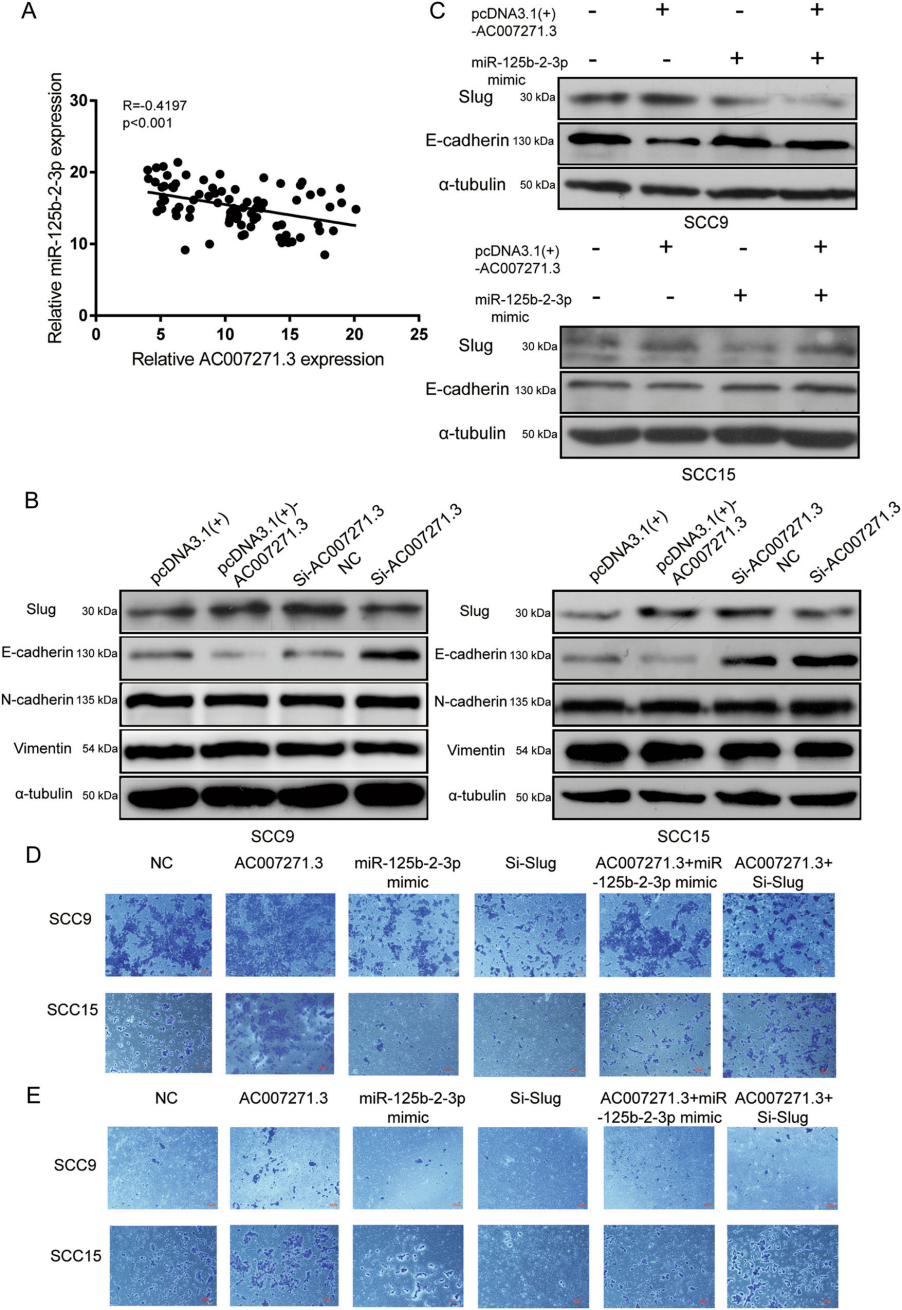
AC007271.3 promoted migration and invasion via miR-125b-2-3p/Slug in OSCC cells. **a** Pearson’s correlation analysis of the relationship between AC007271.3 and miR-125b-2-3p (*n* = 82). **b** Western blot analysis was performed to detect the expression of Slug and EMT‐related markers after overexpressing (or knocking down) AC007271.3 in SCC9 and SCC15. **c** MiR-125b-2-3p mimic could reverse the changing expression of Slug and E-cadherin caused by overexpressed AC007271.3. **d**–**e** Effects of pcDNA3.1(+)-AC007271.3, miR-125b-2-3p mimic, si-Slug on the migration **d** and invasion **e** in SCC9 and SCC15.

The biological function and molecular role of lncRNAs are closely associated with their subcellular localization. We previously identified that AC007271.3 was predominantly located in the cell cytoplasm by using RNA-FISH assay, which indicated that AC007271.3 might function as a ceRNA of miR-125b-2-3p and regulate Slug. To further determine the relationship between AC007271.3, miR-125b-2-3p, and Slug, first, Pearson’s correlation analysis was performed to uncover the association between AC007271.3 and miR-125b-2-3p expression in 82 OSCC tissues. The results indicated that the expression of miR-125b-2-3p was inversely correlated with AC007271.3 (Fig. 5a). Then, the upregulation of Slug and downregulation of E-cadherin were observed when overexpressing AC007271.3, whereas knocking down AC007271.3 resulted in the opposite results (Fig. 5b). In addition, the upregulated Slug and downregulated E-cadherin caused by AC007271.3 was reversed when co-transfecting with miR-125b-2-3p mimic (Fig. 5c). Simultaneously, migration and invasion experiments revealed that miR-125b-2-3p mimic and si-Slug could reverse the effects resulted from AC007271.3 overexpression (Fig. 5d–e).

### 7. Upregulation of AC007271.3 promoted OSCC metastasis in vivo

**Fig. 6:**
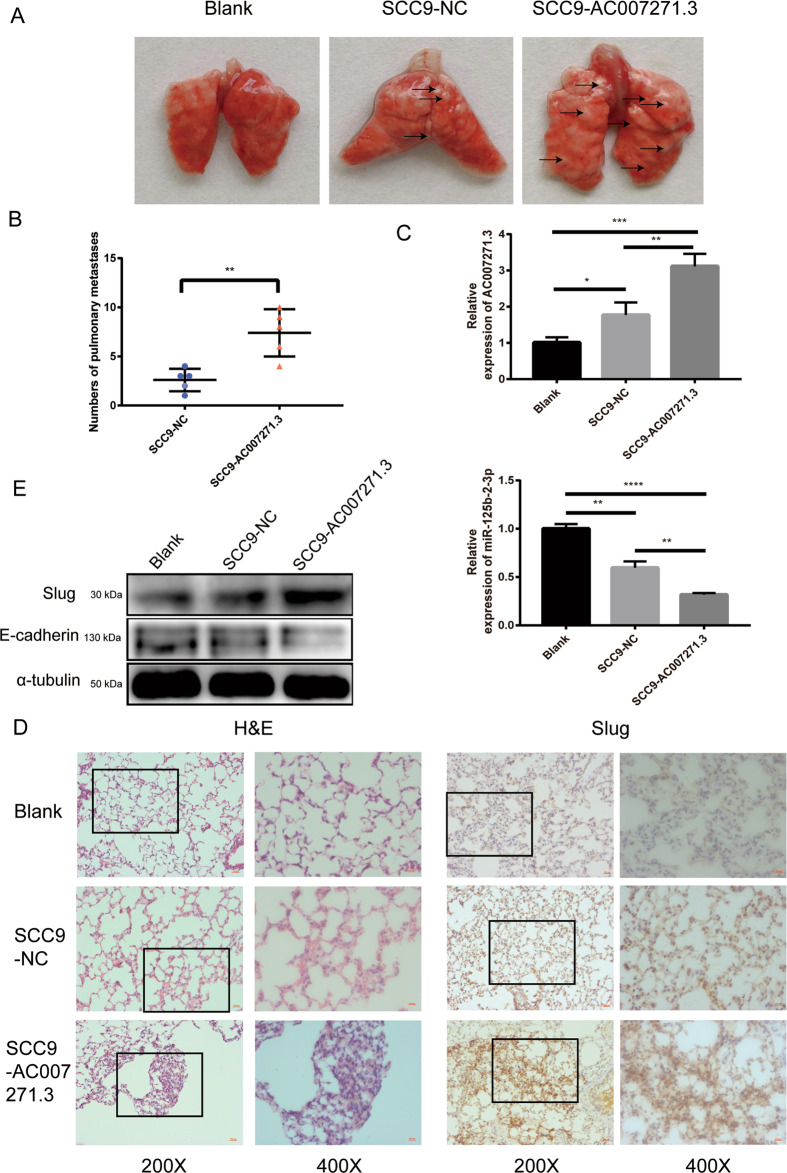
AC007271.3 promoted OSCC metastasis in vivo. **a** Representative images of pulmonary metastatic models. **b** Statistical analysis of colony numbers of pulmonary metastases between SCC9-AC007271.3 and SCC9-NC group (*n* = 5). **c** The expression of AC007271.3 and miR-125b-2-3p in pulmonary metastases were detected by qRT-PCR. **d** Representative images of HE and IHC staining. Higher level of Slug was detected in SCC9-AC007271.3 group than that in SCC9-NC group. **e** Western blot confirmed the upregulation of Slug and the downregulation of E-cadherin in SCC9-AC007271.3 group. *p < 0.05, **p < 0.01, ***p < 0.001, ****p < 0.0001.

A pulmonary metastasis model was established to evaluate the effect of AC007271.3 on OSCC metastasis in vivo. We could clearly see more pulmonary metastatic nodules in SCC9-AC007271.3 group than in SCC9-NC group, which suggests that AC007271.3 could significantly promote OSCC metastasis to lung tissues (Fig. 6a–b). QRT-PCR analysis showed that the expression of AC007271.3 in pulmonary metastases was dramatically increased, whereas miR-125b-2-3p’s RNA level was significantly decreased in the SCC9-AC007271.3 group in contrast to that in the SCC9-NC group (Fig. 6c). Furthermore, H&E staining of lung sections indicated that overexpression of AC007271.3 increased the number and size of pulmonary metastases. The results of IHC staining and western blot also revealed a significant upregulation of Slug in SCC9-AC007271.3 group (Fig. 6d–e). These results indicated that AC007271.3 contributed to OSCC metastasis in vivo.

### 8. The expression of AC007271.3 was promoted by canonical NF-κB pathway

**Fig. 7:**
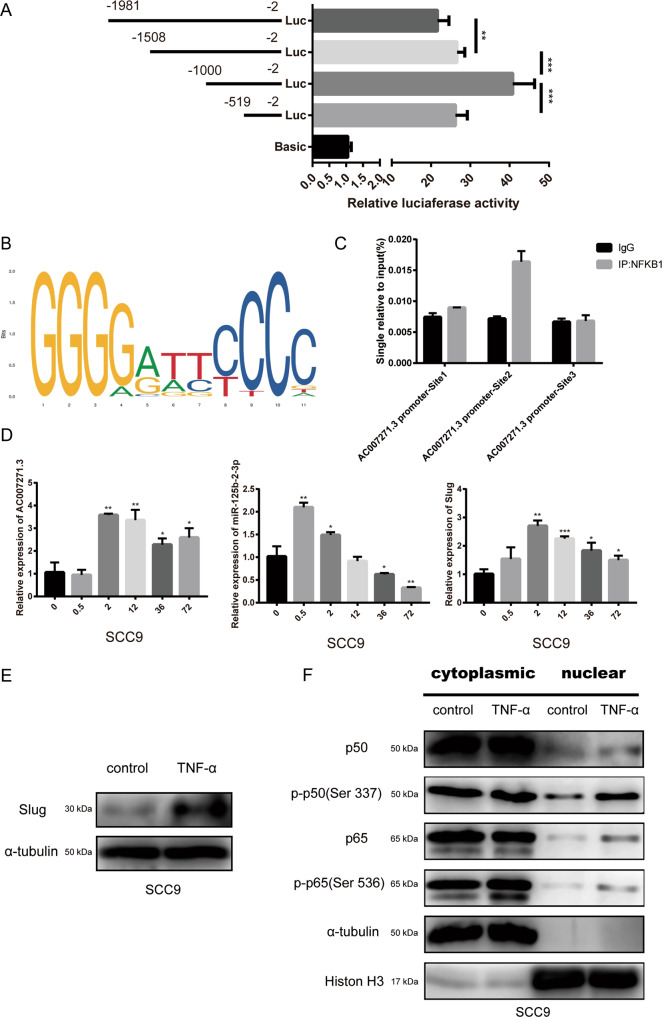
NFKB1 was enriched on the core promoter region of AC007271.3 and the canonical NF-κB pathway upregulated the expression of AC007271.3. **a** After transfecting fragment-by-fragment deletion pGL3 vectors of AC007271.3 promoters into 293 T cells for 48 hours, the luciferase activity was measured and analyzed statistically. **b** The binding sequence logo of NFKB1 on JASPAR database. **c** Enrichment of NFKB1 was detected at the Site 2 (−854/−842) of AC007271.3 core promoter in SCC9 by ChIP-qPCR analysis. **d** After treating with TNF-α (10 ng/ml) for the indicated times in SCC9, the mRNA level of AC007271.3, miR-125b-2-3p, and Slug was analyzed by qRT-PCR. GAPDH was used as a control. **e** The western blot analysis indicated that the protein level of Slug also increased by TNF-α treatment (10 ng/ml, 72 hours). α-tubulin was employed as a control. **f** The canonical NF-κB pathway-related proteins in the cytoplasmic and nuclear of SCC9 which treated with or without TNF-α (10 ng/ml, 72 h) were assessed by western blot. α-tubulin and Histone H3 were employed as the positive controls for cytoplasmic and nuclear proteins, respectively. *p < 0.05, **p < 0.01, ***p < 0.001.

In order to explore the upstream regulation mechanism of AC007271.3, dual-luciferase activity assay was performed for the identification of core promoter region of AC007271.3. The unidirectional deletion at −1000/−519 caused a significant reduction in the luciferase activity (Fig. 7a) so we preliminarily regarded −1000/−519 region as the core promoter of AC007271.3. Next, we predicted that NF-κB may be the potential transcription factor on the core promoter of AC007271.3 on Gene-regulation database. NF-κB is a dimer formed by different kinds of subunits. The prediction on JASPAR database indicated that NFKB1 may be the most critical subunit (Fig. 7b, Supplementary Table S5). To verify our conjecture, we designed three pairs of primers of the predicted binding sites (AC007271.3-promoter Site 1: −953/−943, AC007271.3-promoter Site 2: −854/−842, AC007271.3-promoter Site 3: −581/−571). ChIP-qPCR analysis confirmed that NFKB1 enriched on the AC007271.3-promoter Site 2 (Fig. 7c). NFKB1 is also known as p50, and p50/p65 is the most common type of NF-κB dimer, which is activated by TNF-α in the classical NF-κB pathway and plays a positive regulatory role during gene transcription. Therefore, we measured the mRNA level of AC007271.3, miR-125b-2-3p, and Slug after TNF-α treatment for the indicated times. The mRNA level of AC007271.3 and Slug were stably activated after 0.5 h and 2 h, respectively. On the other hand, interestingly, the mRNA level of miR-125b-2-3p strongly increased after 0.5 h but gradually decreased with the increasing time until 72 h (Fig. 7d). Western blot indicated that the protein level of Slug was also upregulated after TNF-α treatment (Fig. 7e), and at the same time, nuclear translocation of p65 and nuclear-phosphorylation of p50 and p65 were detected (Fig. 7f). These results suggested that the activation of canonical NF-κB pathway could positively regulate AC007271.3 and affect the expression of miR-125b-2-3p and Slug.

## Discussion

Accumulating evidence indicated that lncRNAs are closely related to tumorigenesis and development. Our recent studies confirmed that aberrant AC007271.3 level in OSCC patients was significantly associated with clinical stage, especially in early-stage disease and serum AC007271.3 levels could also discriminate between OSCC and normal controls with high sensitivity and specificity, which suggesting that AC007271.3 could be a novel circulating biomarker for the determination of OSCC^11^. Furthermore, we found that AC007271.3 could promote cell proliferation, invasion, and inhibit cell apoptosis of OSCC via the Wnt/β-catenin signaling pathway, which might provide a novel therapeutic approach for OSCC^12^. In this study, we found that overexpression of AC007271.3 could facilitate the metastasis of OSCC cells in vivo, which further demonstrated the tumorigenesis role of AC007271.3. However, the underlying mechanism of AC007271.3 in OSCC carcinogenesis keep unclear.

LncRNAs’ functions are closely associated with its subcellular localization^22^. Plenty of evidence has indicated that lncRNAs can regulate target genes’ expression by functioning as ceRNAs of miRNAs^15,23^. We previously proved that AC007271.3 was mainly located in the cytoplasm and partly in the nucleus. Thus, it indicated that AC007271.3 may mainly function as an endogenous miRNA sponge to regulate the expression of target genes. In the present study, bioinformatics analysis, luciferase reporter, and RNA pulldown assays revealed that miR-125b-2-3p was a direct target of AC007271.3. Moreover, we interestingly found an inter-regulatory relationship between the expression of AC007271.3 and miR-125b-2-3p. This similar phenomenon was also reported in some researches about ceRNA^17,24^. However, the exact mechanisms were still unknown. In another study, it was reported that lncRNA uc.173 can induce degradation of miR-195 by destabilized its precursor^25^. Therefore, we speculated that whether AC007271.3 could downregulate miR-125b-2-3p through this mechanism and the results confirmed our speculation. To our knowledge, this is the first report to manifest that AC007271.3 could not only played as a ceRNA of miR-125b-2-3p, but also regulated miR-125b-2-3p by destabilized pri-miR-125b-2 in OSCC.

MiR-125b-2-3p is a member of human miR-125 family, which consists of three homologs (miR-125a, miR-125b-1, and miR-125b-2). The miR-125b-2-3p was derived from the 3 ‘arm of the precursor miRNA(pre-miRNA) of miR-125b-2^26^. It had reported that miR-125b-2-3p was downregulated in hepatocellular carcinoma^27^ and small cell osteosarcoma^28^ but upregulated in tumor stroma of colon cancer^29^. In OSCC, although miR-125b was found to inhibit the progression of OSCC^30^, the function of miR-125b-2-3p is still uncertain. In this study, we predicted and confirmed that miR-125b-2-3p directly regulated Slug to function as a OSCC tumor suppressor.

Slug, also known as Snail Family Transcriptional Repressor 2 (Snai2), is a member of the Snail family (Snai1, Snai2, Snai3). It is an important transcription factor closely relates to the occurrence and development of various diseases including cancer^31^. Aberrant expression of Slug has been closely related to cancer stem cell formation, cell cycle regulation, and apoptosis as well as invasion and metastasis^21^. As we predicted in the present study, Slug was a target gene of miR-125b-2-3p by bioinformatics analysis and then verified by luciferase reporter assays and western blot. The expression of Slug was remarkably upregulated in OSCC tissues than in normal controls and meant unfavorable prognosis by bioinformatics analysis. We further found that Slug expression was significantly higher in OSCC tissues than in the ANTs by immunohistochemistry and high expression of Slug was positively correlated with TNM classification, lymph node metastasis, and differentiation in OSCC patients. In addition, knockdown of Slug expression remarkably impaired the ability of migration and invasion of OSCC cells. These results discovered that Slug might play an important role in OSCC carcinogenesis. Furthermore, we found that AC007271.3 positively regulated the expression of Slug. In the rescue experiment, overexpression of miR-125b-2-3p reversed the upregulated Slug caused by overexpressed AC007271.3. Simultaneously, migration and invasion experiments unveiled that miR-125b-2-3p mimic and si-Slug can reverse the effects resulted from AC007271.3 overexpression. Taken together, AC007271.3 could regulate Slug expression by regulating miR-125b-2-3p.

Snai family members could directly bind to the promoter of E-cadherin by E-box region and negatively regulate its expression^32,33^. E-cadherin, N-cadherin, and vimentin were the frequent symbol markers for EMT^34^, which could enhance the invasion and migration of tumors. In this work, we found that the knockdown of Slug induced no significant changes on N-cadherin, vimentin, and β-catenin except a remarkable decrease of E-cadherin, which indicated that Slug could change the EMT phenotype by inhibiting the expression of E-cadherin and then promoted the migration and invasion in OSCC cells. On the other hand, the E-cadherin/catenin complex, which binds to cytoskeletal components, is an important regulator to form a mature adherent junction^35^. The reduction in E-cadherin was related to the activating of β-catenin in colorectal cancer^36^. Combined with our results, we speculated that Slug may increase the dissociated β-catenin, which could be activated and translocated into the nucleus, by reducing the expression of E-cadherin. Moreover, recent researches implied that the Wnt/β-catenin pathway participated in the regulation of Slug^37,38^, which may form a feed-back regulation between Slug/E-cadherin/β-catenin. However, the relation between Slug, E-cadherin, and β-catenin in OSCC needs to be further explored.

NF-κB is a regulator of expression of the κB light chain firstly recognized in B cells. It is a homo- or heterodimer formed by any two of the five subunits of RelA (p65), RelB, c-Rel, NFKB1 (p50), NFKB2 (p52)^39^. Plenty of evidence indicated that NF-κB pathway played an important role in inflammation and cancer^40,41^. In the canonical NF-κB pathway, tumor necrosis factor (TNF)-α stimulation induces IKKβ phosphorylation and then phosphorylates IκBα to promote its polyubiquitination and degradation, which lead to the release of p65/p50 heterodimer. P65/p50 dimers may be phosphorylated^42^ and then translocate into the nucleus and bind to the specific DNA sequences to promote the transcription of target genes^43^. The NF-κB pathway has been fully studied in the past few decades. It was widely acknowledged that p50/p65 dimer plays a direct role in the canonical NF-κB pathway to positively regulate the transcription of target genes^39–41,43^. Phosphorylation sites of p50 and p65 also regulate the function of this dimer^42,44^. Previous studies showed that p50 (Ser337) site phosphorylation could enhance the DNA binding ability of p50^45^, whereas p65 (Ser536) phosphorylation could enhance the transactivation potential of p65^46^. In the research, after treatment of TNF-α in SCC9, the overexpressed AC007271.3 were detected, and the p50 (Ser337) and p65 (Ser536) were significantly upregulated in the nucleus. These results suggested that the activation of the canonical NF-κB pathway could positively regulate the expression of AC007271.3. In addition, a recent study showed that NF-κB pathway promoted metastasis in head and neck squamous cell carcinoma cells by stabilizing Slug^47^. However, the underlying mechanism is not clear. Our results identified that the classical NF-κB pathway probably regulated the migration and invasion of OSCC through the AC007271.3/miR-125b-2-3p/Slug axis. To our knowledge, it is the first time to propose this potential mechanism in OSCC.

## Conclusion

In conclusion, our research confirmed that the classical NF-κB pathway-regulated AC007271.3 interacted with miR-125b-2-3p to regulate Slug gene and promote the migration and invasion in OSCC cells, which suggested that AC007271.3 may be a diagnostic molecule and therapeutic target for OSCC.

## Supplementary information

Supplementary Table

Supplementary Table Legends

Supplementary Figure S1

Supplementary Figure S2

Supplementary Figure S3

Supplementary Figure S4

Supplementary Figure Legends
