# Supplementary material for: NF-κB-mediated lncRNA AC007271.3 promotes carcinogenesis of oral squamous cell carcinoma by regulating miR-125b-2-3p/Slug
Source: Cell Death Dis. 2020 Dec 12;11(12):1055. doi: 10.1038/s41419-020-03257-4 (PMC7733441; doi:10.1038/s41419-020-03257-4)
Supplement: Supplementary file 1 — Supplementary Table [file 41419_2020_3257_MOESM1_ESM.docx]

**Supplementary Table S1**

Primer information of construction of segment-by-segment deletion of the promoter region of AC007271.3 and qPCR (includes ChIP-qPCR) analysis.

| **Primer** | **Sequence (5’ to 3’)** |
| --- | --- |
| AC007271.3-F | ACGTACTTTGAGCAGCCAAG |
| AC007271.3-R | ACCTTCTGCAGAGAGTTCTTCC |
| GADPH-F | TGAACGGGAAGCTCACTGG |
| GADPH-R | TCCACCACCCTGTTGCTGTA |
| Slug-F | TGTGACAAGGAATATGTGAGCC |
| Slug-R | TGAGCCCTCAGATTTGACCTG |
| U6-F | CTCGCTTCGGCAGCACA |
| U6-R | AACGCTTCACGAATTTGCGT |
| miR-125b-2-3p-F | CGCGTCACAAGTCAGGCTCT |
| miR-125b-2-3p-R | AGTGCAGGGTCCGAGGTATT |
| miR-1301-3p-F | TTGCAGCTGCCTGGGAGT |
| miR-1301-3p-R | AGTGCAGGGTCCGAGGTATT |
| miR-4801-F | GCGGCGGTACACAAGAAAACCAAG |
| miR-4801-R | AGTGCAGGGTCCGAGGTATT |
| pri-miR-125b-2-F | ACCAGACTTTTCCTAGTCCCTG |
| pri-miR-125b-2-R | CCCAAGAGCCTGACTTGTGA |
| AC007271.3(-1981)-F | GGGGTACCCCCTGAGTGCCAGAAATAC |
| AC007271.3(-1508)-F | GGGGTACCCCTCCTGGCTTCAAGTGAT |
| AC007271.3(-1000)-F | GGGGTACCCCTTTAGGTTGTGAAGTTATGGGTG |
| AC007271.3(-519)-F | GGGGTACCCCAAGTACGCTTGAGAACA |
| AC007271.3(-2)-R | GAAGATCTTCCAGGGTGAAGTTGATGAAGC |
| NFKB1-ChIP-S1-F | TTAGGTTGTGAAGTTATGGGTG |
| NFKB1-ChIP-S1-R | GAAAGCAATAATGGTTTGAGAG |
| NFKB1-ChIP-S2-F | AACCATTATTGCTTTCGTA |
| NFKB1-ChIP-S2-R | AGAAACACATTGATAGGGC |
| NFKB1-ChIP-S3-F | TCCAAATAGTTCAGTCA |
| NFKB1-ChIP-S3-R | AAAAGTGCTAACCAAG |

**Supplementary Table S2**

Primary antibodies used in this study.

| **Antibody(item NO.)** | **Antibody dilution** | | | **Specificity** | **Company** |
| --- | --- | --- | --- | --- | --- |
|  | **WB** | **IHC** | **ChIP** |  |  |
| Slug(9585) | 1:1000 |  |  | Rabbit | Cell Signaling Technology |
| Slug(A1057) |  | 1:50 |  | Rabbit | Abclonal Technology |
| α-tubulin(AC012) | 1:1000 |  |  | Mouse | Abclonal Technology |
| N-cadherin(A3045) | 1:1000 |  |  | Rabbit | Abclonal Technology |
| E-cadherin(A11509) | 1:1000 |  |  | Rabbit | Abclonal Technology |
| β-catenin(8814) | 1:1000 |  |  | Rabbit | Cell Signaling Technology |
| Vimentin(10366-1-AP) | 1:2000 |  |  | Rabbit | Proteintech Group |
| NFKB1(13586) | 1:1000 |  | 1:50 | Rabbit | Cell Signaling Technology |
| phospho-NFKB1-S337(AP0125) | 1:1000 |  |  | Rabbit | Abclonal Technology |
| p65(8242) | 1:1000 |  |  | Rabbit | Cell Signaling Technology |
| Phospho-p65-Ser536(3033) | 1:1000 |  |  | Rabbit | Cell Signaling Technology |
| Histone H3(4499) | 1:2000 |  |  | Rabbit | Cell Signaling Technology |

**Supplementary Table S3**

Correlation between the expression levels of miR-125b-2-3p and clinicopathological features in 82 OSCC patients

| **Feathers** | **Number** | **High** | **Low** | **P value** |
| --- | --- | --- | --- | --- |
| All cases | 82 | 41 | 41 |  |
| Age, years  ＜60  ≥60 | 33  49 | 16  25 | 18  23 | 0.8228 |
| Gender  Male  Female | 56  26 | 26  15 | 30  11 | 0.4769 |
| TNM classification  Ⅰ and Ⅱ  Ⅲ and Ⅳ | 34  48 | 22  19 | 12  29 | **0.0429^*^** |
| Lymphatic metastasis  N_0_  N_1_-N_3_ | 37  45 | 24  17 | 13  28 | **0.0259^*^** |
| Differentiation  Well/moderate  Poor | 51  31 | 28  13 | 23  18 | 0.3625 |

Abbreviations: OSCC oral squamous cell carcinoma, TNM tumor-nodes-metastases, * statistically significant

**Supplementary Table S4**

Correlation between the expression levels of Slug and clinicopathological features in 82 OSCC patients

| **Feathers** | **Total** | **High Slug expression** | **Low Slug expression** | **P value** |
| --- | --- | --- | --- | --- |
| Cases | 82 | 57 | 25 |  |
| Age, years  ＜60  ≥60 | 33  49 | 22  35 | 11  14 | 0.8071 |
| Gender  Male  Female | 56  26 | 38  19 | 18  7 | 0.7975 |
| TNM classification  Ⅰ and Ⅱ  Ⅲ and Ⅳ | 34  48 | 19  38 | 15  10 | **0.0301^*^** |
| Lymphatic metastasis  N_0_  N_1_-N_3_ | 37  45 | 22  35 | 15  10 | **0.0306^*^** |
| Differentiation  Well/moderate  Poor | 51  31 | 31  26 | 20  5 | **0.0465^*^** |

Abbreviations: OSCC oral squamous cell carcinoma, TNM tumor-nodes-metastases, * statistically significant

**Supplementary Table S5**

Prediction of the binding motifs of NF-κB in AC007271.3 core promoter region.

| Transcription factor | Matrix ID | Predicted sequence | Score | Relative score | Start | End |
| --- | --- | --- | --- | --- | --- | --- |
| NFKB1 | MA0105.3 | AGAAATTTCCA | 9.26642 | 0.884157159825 | -953 | -943 |
| NFKB2 | MA0778.1 | AGGGGATTACTCT | 8.75264 | 0.856121652552 | -854 | -842 |
| NFKB1 | MA0105.4 | AGGGGATTACTCT | 7.30992 | 0.839333491651 | -854 | -842 |
| NFKB1 | MA0105.1 | GGGGATTACT | 8.23833 | 0.835067559571 | -853 | -844 |
| NFKB1 | MA0105.3 | TGTAATTTTCT | 5.27985 | 0.825998998156 | -581 | -571 |
| NFKB1 | MA0105.2 | GGGGATTACTC | 8.74924 | 0.811908132548 | -853 | -843 |
| REL | MA0101.1 | GGGGATTACT | 6.51267 | 0.802215524335 | -853 | -844 |

Abbreviations: NFKB1 Nuclear Factor Kappa B Subunit 1 (p50), NFKB2 Nuclear Factor Kappa B Subunit 2 (p52), Rel REL Proto-Oncogene, NF-KB Subunit
