# Supplementary material for: NF-κB-mediated lncRNA AC007271.3 promotes carcinogenesis of oral squamous cell carcinoma by regulating miR-125b-2-3p/Slug
Source: Cell Death Dis. 2020 Dec 12;11(12):1055. doi: 10.1038/s41419-020-03257-4 (PMC7733441; doi:10.1038/s41419-020-03257-4)
Supplement: Supplementary file 2 — Supplementary Table Legends [file 41419_2020_3257_MOESM2_ESM.docx]

**Supplementary Table S1**

Primer information of construction of segment-by-segment deletion of the promoter region of AC007271.3 and qPCR (includes ChIP-qPCR) analysis.

**Supplementary Table S2**

Primary antibodies used in this study.

**Supplementary Table S3**

Correlation between the expression levels of miR-125b-2-3p and clinicopathological features in 82 OSCC patients.

**Supplementary Table S4**

Correlation between the expression levels of Slug and clinicopathological features in 82 OSCC patients.

**Supplementary Table S5**

Prediction of the binding motifs of NF-κB in AC007271.3 core promoter region.
