# Supplementary figures and images for: NF-κB-mediated lncRNA AC007271.3 promotes carcinogenesis of oral squamous cell carcinoma by regulating miR-125b-2-3p/Slug
Source: Cell Death Dis. 2020 Dec 12;11(12):1055. doi: 10.1038/s41419-020-03257-4 (PMC7733441; doi:10.1038/s41419-020-03257-4)

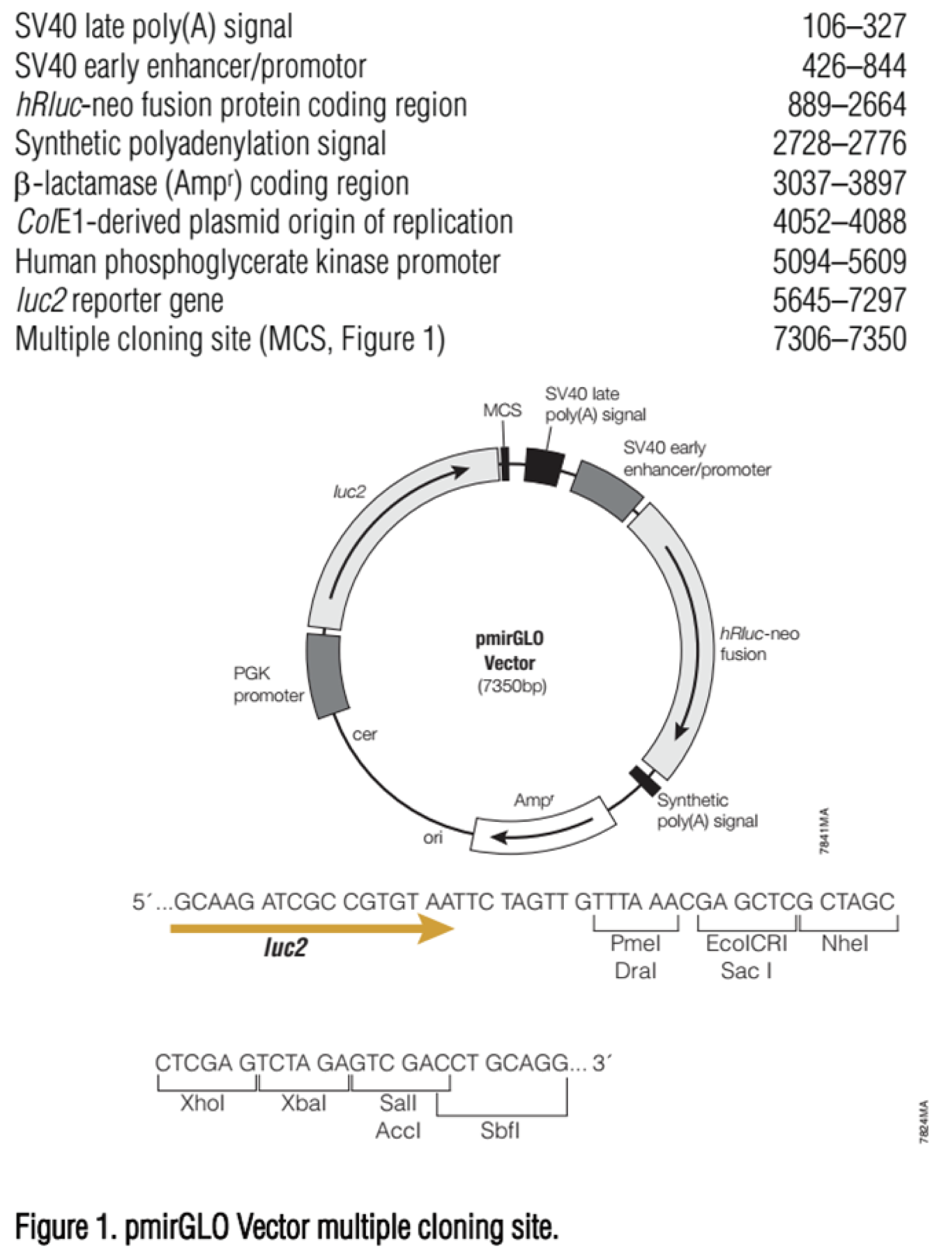

Supplement: Supplementary file 3 — Supplementary Figure S1 [file 41419_2020_3257_MOESM3_ESM.png]

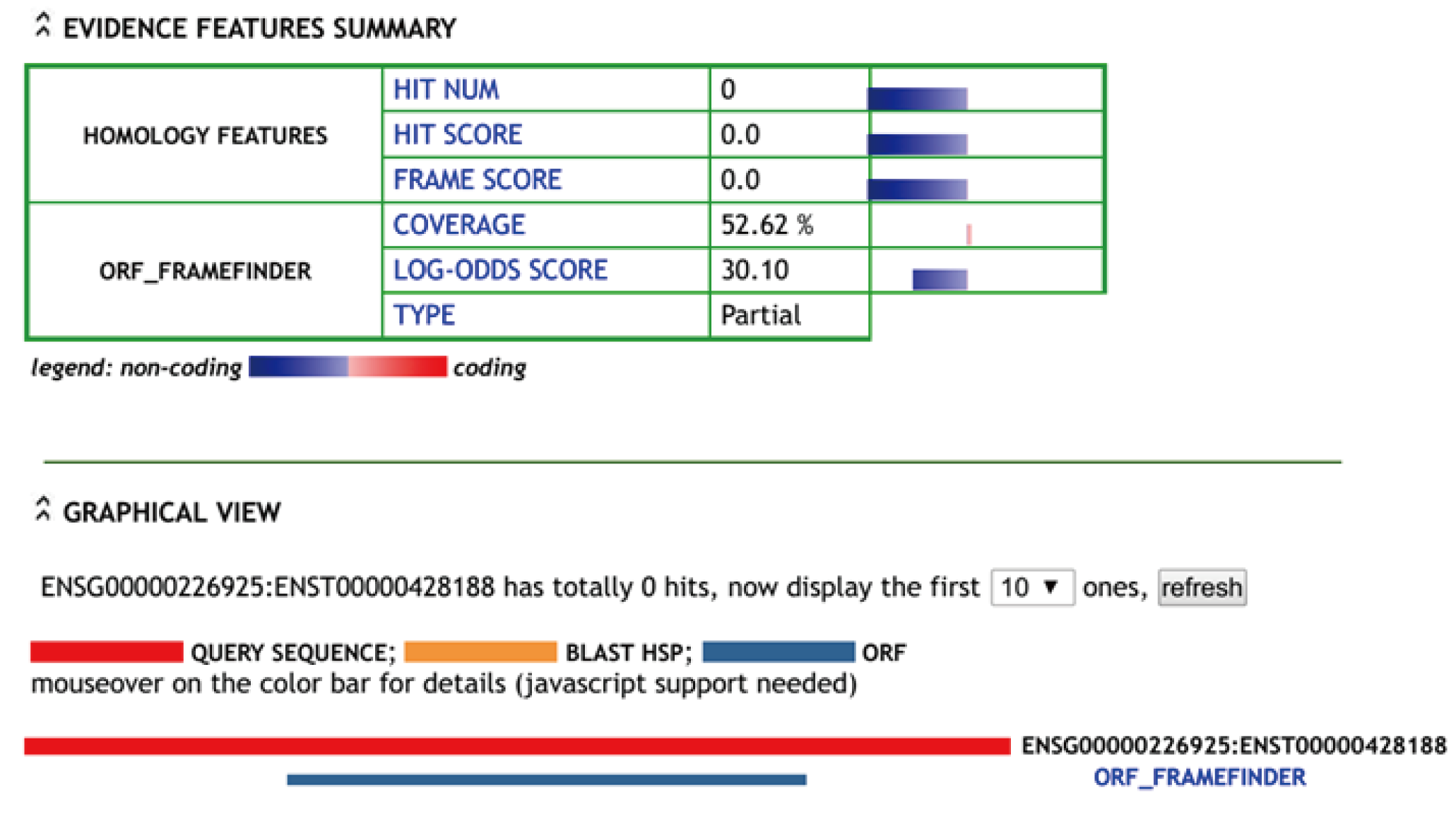

Supplement: Supplementary file 4 — Supplementary Figure S2 [file 41419_2020_3257_MOESM4_ESM.png]

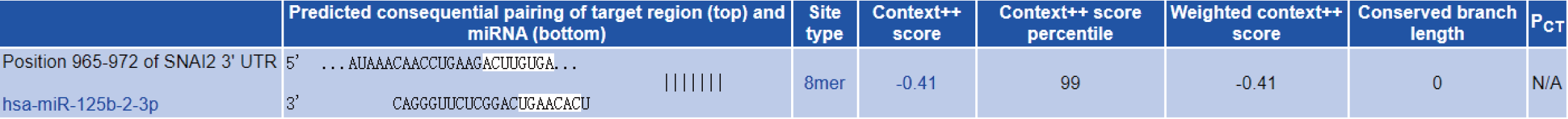

Supplement: Supplementary file 5 — Supplementary Figure S3 [file 41419_2020_3257_MOESM5_ESM.png]

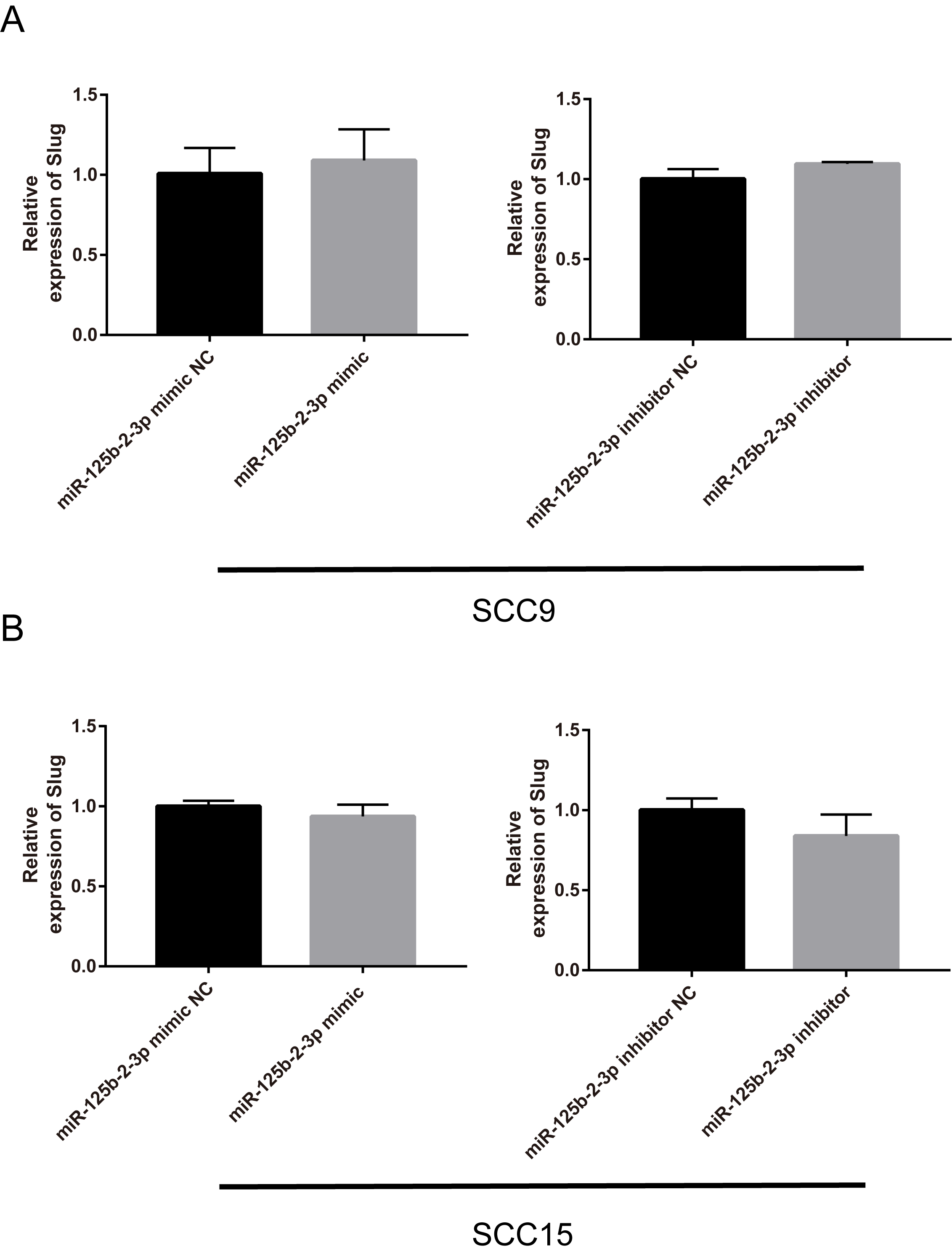

Supplement: Supplementary file 6 — Supplementary Figure S4 [file 41419_2020_3257_MOESM6_ESM.png]
