# Supplementary material for: NF-κB-mediated lncRNA AC007271.3 promotes carcinogenesis of oral squamous cell carcinoma by regulating miR-125b-2-3p/Slug
Source: Cell Death Dis. 2020 Dec 12;11(12):1055. doi: 10.1038/s41419-020-03257-4 (PMC7733441; doi:10.1038/s41419-020-03257-4)
Supplement: Supplementary file 7 — Supplementary Figure Legends [file 41419_2020_3257_MOESM7_ESM.docx]

**Supplementary Figure S1**

The features list and map for the pmirGLO Vector showed in its product instruction.

**Supplementary Figure S2**

The prediction of AC007271.3 protein coding ability.

**Supplementary Figure S3**

The predicted binding sites between miR-125b-2-3p and 3’UTR of Slug mRNA on TargetScan database.

**Supplementary Figure S4**

No significant change of Slug mRNA was observed after overexpressing or inhibiting miR-125b-2-3p in SCC9 (a) and SCC15 (b).
